# Effects of synchronised engine sound and vibration presentation on visually induced motion sickness

**DOI:** 10.1038/s41598-020-64302-y

**Published:** 2020-05-12

**Authors:** Yuki Sawada, Yoshihiro Itaguchi, Masami Hayashi, Kosuke Aigo, Takuya Miyagi, Masayuki Miki, Tetsuya Kimura, Makoto Miyazaki

**Affiliations:** 1 Department of Computer Science, Faculty of Informatics, Shizuoka University, Hamamatsu, Japan; 2 Department of Informatics, Graduate School of Integrated Science and Technology, Shizuoka University, Hamamatsu, Japan; 3 Yamaha Motor Co., Ltd., Iwata, Japan

**Keywords:** Sensorimotor processing, Sensory processing, Human behaviour

## Abstract

Driving simulator usage is often accompanied by motion sickness, and techniques for its prevention are not yet established. To reduce visually induced motion sickness (VIMS), we investigated the effects of synchronised presentation of engine sounds and motorcycle vibration on VIMS. A total of 80 participants experienced a driving scene with a head-mounted display for 5 minutes with or without synchronised presentation of engine sound and vibration. The results showed that VIMS scores, as measured by the Fast Motion Sickness scale, were significantly lower in participants who experienced the driving scene with sounds and vibration than in those who experienced the scene with sounds only, vibration only, or neither. Multiple regression analyses revealed that susceptibility to VIMS consistently explained the severity of VIMS to some extent but not with perceived realism of the virtual reality (VR) scene, sex, and experiences about VR devices and vehicles. This study demonstrated that simultaneous presentation of engine sounds and vibration, which were synchronous to each other and tightly coupled with the visual flow speed, effectively reduces VIMS while experiencing motorcycling simulators. The findings not only improve practical knowledge for reducing VIMS in driving simulators but also provide evidence for understanding the mechanisms of VIMS.

## Introduction

Driving simulators^1^ let their users experience virtual driving scenes, which is useful in many applications, including the safe evaluation and improvement of driving abilities^2–5^. However, driving simulators can cause simulator sickness, which is one type of visually induced motion sickness (VIMS)^6,7^. VIMS is a broad concept, including cybersickness and virtual reality (VR) sickness, and is elicited at a probability of 30–80%^8^. Although the frequency and magnitude of VIMS depend on simulator types and driving tasks, VIMS can be elicited regardless of the availability of a motion system (fixed-base or moving-base) and the type of displays. The severity of the sickness increases by the length of visual scene observation^7,9,10^. VIMS is characterised by various symptoms such as drowsiness, dizziness, fatigue, cold sweat, headache, nausea, stomach discomfort, pallor and vomiting^11–13^. Although previous studies have tried to reveal the mechanism of VIMS using extensive types of visual stimuli, such as driving scenes^3,4,14^ and VR games^15,16^, a consensus has not been reached about what causes VIMS. Furthermore, some of the currently proposed techniques (e.g., narrowing the field of view) to reduce VIMS could deteriorate the VR experience, or the effect size was not reliable^17^; therefore, immediate implementation of the techniques has been hindered. In this study, we examined the effect of synchronised presentation of engine sound and vibration on VIMS to develop more comfortable driving simulators in a simple and economical way.

The most influential theory for explaining the mechanism of motion sickness is the sensory conflict theory^18^, which, however, has been loosely defined and has many variants in the literature^11^. The sensory conflict theory originally assumes that motion sickness is caused by a mismatch between sensory inputs between modalities. Reason^18^ introduced the idea of a neural store, which represents the memory of paired sensory input and motor command, and thus neural mismatch signals are proportional to the magnitude of a difference between the expected sensory input and actual sensory input^11^. Although Reason^18^ and Oman^11^ mainly focused on the sensory conflict with vestibular inputs, they also suggest that conflicts between visual and other modality inputs without vestibular conflicts can provoke motion sickness. According to this theory, motion sickness associated with a driving simulator is induced by the inconsistency between the expected sensory inputs based on a real-world experience and actual sensory inputs in terms of sensory correspondence of visual, auditory, somatosensory and vestibular sensory information.

Various factors have been demonstrated to be involved in VIMS. Draper, *et al*.^19^ showed that more than 48 ms of display delay in a head-mounted display (HMD) drastically increased the occurrence of VIMS, which has impacted the guideline of HMD developers like Oculus (Facebook technologies, LLC., USA), which requires the display delay (motion-to-photon latency) to be less than 20 ms. The motion sickness is more likely to be induced by 3-dimensional than by 2-dimensional visual stimuli^20^. Display resolution, field of view and angular velocity of the visual stimulus are also known factors associated with VIMS^19,21^. Individual characteristics such as sex^15^, age ^5,22^, stress and driving experience^23^, as well as sleep problems^24^ may affect the severity or probability of VIMS. In addition, VIMS could be reduced by the adaptation for repetitive experiences^10,25,26^, narrowing the field of view ^17,27^, showing a “nose” in the visual field as a reference^28^, bone-conducted vibration to the vestibular system^29^ and a comfortable music and smell^30^.

Usually, it is not easy to avoid motion sickness when one watches a moving scene on a display that has a large field of view. D’Amour, *et al*.^31^ investigated the effects of vibration and airflow on VIMS. In their experiment, participants watched a visual scene from a first-person view that one is driving a bicycle in a town. They found a significant reduction effect only of airflow, but not of vibration, on VIMS. In addition, Keshavarz and Hecht did not found significant reduction effects either of background sounds during a bicycle ride^20^, nor of environmental noises, footsteps or the character’s breathing during a video game from the first-person perspective^32^. These findings suggest that airflow, but not a simple presentation of sounds and vibration, is effective in reducing VIMS at least in experiencing a VR scene of a bicycle ride, which usually runs against the wind. Furthermore, a recent study of Keshavarz, *et al*.^5^ reported that presenting engine sounds, motion cues and their combination did not reduce VIMS in a car driving simulator.

Nevertheless, in a motorcycle driving scene, it is expected that synchronously presented engine sounds and vibration have substantial effects on VIMS reduction. The sensory conflict theory argues that VIMS will not happen if anticipated sensory integration occurs or multisensory integration is successfully resolved^7^; therefore, the presentation of more than one modality of sensory inputs that coincide in reality would decrease VIMS. Accordingly, it would be possible that synchronised presentation of engine sounds and vibration, which are always coupled and proportional to engine speed in reality, decreases VIMS, although the simple presentation of them cannot reduce VIMS. A simple addition of individual sensory feedback could rather elicit a “conflict” to the stored sensory integration because the corresponded feedbacks are missing. This mechanism is possibly linked to the “uncanny valley” in the humanoid literature. In the study by D’Amour, *et al*.^31^, the simultaneous presentation of airflow and vibration did not decrease VIMS compared with a simple presentation of airflow. This result could be explained by assuming that the addition of information is not coupled in reality, and the vibration is constant regardless of the driving scene or airflow. The insignificant effect of background noise^20,32^ can be explained by introducing the idea that the motion of the visual field and background sounds were not sufficiently coupled; that is, the sounds such as the noise of the town might not be helpful to predict changes in the first person’s view. The insignificant effects of combining speed-dependent engine sounds and motion cues on VIMS in a car driving simulator^5^ might be due to the lack of tight coupling among engine sounds, motion cues and the visual flow, although the motion information was not clear from the study description. The present study thus focused on the sounds and vibration both induced by an engine, which are inevitably accompanied by a motorcycle driving.

In this study, we specifically investigated whether a synchronised presentation of engine sounds and vibration decreases VIMS while watching a motorcycle-driving scene in a virtual environment. The engine sounds and vibration were modulated by motorcycle driving speed. Participants sat on a chassis of a scooter and wore an HMD, and vibration was provided to participants under the seat. An HMD has advantages in presenting an immersive visual environment without large-scaled equipment and in installing dynamic interactions easily ^33,34^. In addition, applying vibration has an advantage in installing to driving simulators because the system can be established with consumer devices, and the algorithm is not special. We used the Simulator Sickness Questionnaire (SSQ)^35^ and Fast Motion Sickness (FMS) scale^9^ to evaluate motion sickness based on subjective reports^31^. SSQ is a questionnaire to evaluate the degree of motion sickness subjectively, and FMS was invented to quickly evaluate the degree of motion sickness by verbally reporting the number. Because previous studies have suggested other individual factors such as realism and presence of virtual environment and past experiences about car, motorcycle and VR could affect VIMS ^23,34,36,37^, we additionally analysed these factors using multiple linear regression.

## Results

### FMS and SSQ scores

**Figure 1:**
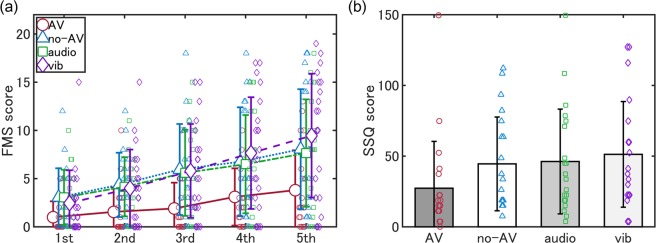
FMS and total SSQ scores. FMS scores are significantly lower in the AV group than in the other groups. SSQ scores are not significantly different among the groups. FMS: Fast Motion Sickness scale; SSQ: Simulator Sickness Questionnaire; AV: Audio-vibration; Vib: Vibration.

The participants (n = 80) were randomly assigned to the audio-vibration group (AV group), no-audio-vibration group (no-AV group), audio-only group, or vibration-only group. They experienced a 5-minute motorcycle driving scene. The average FMS scores during watching a driving scene are shown in Fig. 1a and total SSQ scores after watching the scene are presented in Fig. 1b. The average scores related to VIMS were summarised in Table 1. We used a two-factor ANOVA (4 experimental groups × 5 evaluation phases) instead of a three-way ANOVA (audio presentation × vibration presentation × evaluation phase) to focus on the effect of simultaneous audio-vibration presentation on VIMS reduction.

**Table 1:** Average values of the VIMS-related variables, realism and presence scores.

			FMS 1st	FMS 2nd	FMS 3rd	FMS 4th	FMS 5th	SSQ-TS	SSQ-O	SSQ-N	SSQ-D	MSSQ	Realism	Presence
Total	AV	Mean	1.0	1.6	1.9	3.1	3.8	27.3	2.6	3.0	1.8	77.2	5.6	6.6
		SD	1.7	2.6	2.8	3.0	4.2	34.0	2.7	3.6	3.2	30.2	2.4	2.2
	no-AV	Mean	3.1	4.4	6.0	6.8	8.1	44.5	3.5	4.9	3.6	80.5	4.1	6.1
		SD	3.1	3.4	4.8	5.8	6.4	34.0	2.9	4.4	2.9	25.7	1.5	2.2
	audio-only	Mean	3.1	4.2	5.6	6.5	7.7	46.2	4.0	5.6	2.8	70.5	6.1	6.9
		SD	2.9	3.2	4.6	5.2	5.7	38.0	3.0	4.4	3.3	18.9	1.4	1.5
	vib-only	Mean	2.5	4.0	5.8	7.7	9.5	51.2	4.7	5.8	3.3	91.3	6.1	7.1
		SD	3.5	4.1	5.0	5.9	6.6	38.3	3.4	4.5	2.9	32.9	1.8	1.9
Male	AV	Mean	0.4	0.8	1.3	2.2	2.4	16.8	1.7	1.9	0.9	74.2	5.6	6.4
		SD	0.9	1.2	1.9	2.2	2.8	13.0	1.1	1.6	1.7	27.1	2.4	2.1
	no-AV	Mean	3.3	4.5	6.1	6.8	8.2	44.6	3.6	4.8	3.5	79.2	4.1	5.9
		SD	3.3	3.6	5.3	6.2	6.6	36.8	3.0	4.7	3.3	25.6	1.2	2.2
	audio-only	Mean	3.1	4.2	5.6	6.4	7.6	47.7	4.1	5.8	2.9	67.2	6.0	6.8
		SD	3.2	3.3	4.7	4.8	5.0	39.6	3.0	4.6	3.6	17.4	1.3	1.3
	vib-only	Mean	2.6	4.1	5.7	7.6	9.5	52.6	4.8	5.8	3.5	90.1	6.1	6.9
		SD	3.8	4.1	4.8	5.8	6.4	35.1	2.9	4.3	2.7	34.5	1.7	1.9
Female	AV	Mean	3.5	4.8	4.5	6.8	9.3	69.2	6.3	7.3	5.0	89.0	5.8	7.0
		SD	1.9	4.3	4.2	3.4	5.0	59.6	4.2	6.1	5.7	43.0	2.6	2.9
	no-AV	Mean	2.0	3.8	5.5	6.5	7.5	43.9	2.8	5.3	3.8	85.8	4.0	6.5
		SD	2.4	3.0	3.1	4.4	6.4	23.9	2.9	3.9	0.5	29.3	2.4	2.4
	audio-only	Mean	2.8	4.0	5.5	7.0	8.0	40.2	3.3	5.0	2.5	83.6	6.3	7.0
		SD	2.1	2.9	4.7	7.6	8.9	35.1	3.3	3.9	2.4	21.5	2.1	2.2
	vib-only	Mean	2.0	3.8	6.3	8.0	9.3	45.8	4.3	5.8	2.3	96.3	6.0	7.8
		SD	2.4	4.8	6.5	7.3	8.4	55.6	5.3	5.7	3.9	29.3	2.4	2.2

FMS: Fast Motion Sickness scale; SSQ: Simulator Sickness Questionnaire; AV: audio-vibration; vib: vibration; TS: total score; N: nausea; O: oculomotor; D: disorientation.

The two-factor ANOVA on FMS scores revealed significant main effects of the experimental group (*F* [3, 76] = 3.63, *p* = 0.02, *ω*^2^ = 0.06) and evaluation period (*F* [4, 304] = 58.64, *p* < 0.001, *ω*^2^ = 0.12), and a significant interaction effect of experimental group and evaluation period (*F* [12, 304] = 1.95, *p* = 0.03, *ω*^2^ = 0.01). Simple main effects of experimental group were significant in all periods except for the 1^st^ period (1^st^: *F* [3, 76] = 2.24, *p* = 0.09, *ω*^2^ = 0.04; 2^nd^: *F* [3, 76] = 3.05, *p* = 0.03, *ω*^2^ = 0.07; 3^rd^: *F* [3, 76] = 3.94, *p* = 0.01, *ω*^2^ = 0.10; 4^th^: *F* [3, 76] = 3.02, *p* = 0.03, *ω*^2^ = 0.07; 5^th^: *F* [3, 76] = 3.47, *p* = 0.02, *ω*^2^ = 0.08), and simple main effects of evaluation period were significant in all groups (AV: *F* [4, 76] = 11.16, *p* < 0.001, *ω*^2^ = 0.10; no-AV: *F* [4, 76] = 13.66, *p* < 0.001, *ω*^2^ = 0.11; audio-only: *F* [4, 76] = 11.08, *p* < 0.001, *ω*^2^ = 0.11; vibration-only: *F* [4, 76] = 25.26, *p* < 0.001, *ω*^2^ = 0.18). Multiple comparisons revealed that from the 2^nd^ period, there were significant differences in FMS between the AV group and other groups. Table 2 indicates the statistical results of the multiple comparisons. Note that we compared only pairs between the AV group and other groups using Holm’s method, considering the focus of the present study and to keep the statistical power in the analysis.

**Table 2:** The results of multiple comparisons between the AV group and other groups in each evaluation phase.

	FMS 1st			FMS 2nd			FMS 3rd			FMS 4th			FMS 5th		
	*t*	non-adj. *p*		*t*	non-adj. *p*		*t*	non-adj. *p*		*t*	non-adj. *p*		*t*	non-adj. *p*	
AV vs no-AV	2.24	0.028	*n.s*.	2.63	0.010	*	2.92	0.006	*	2.25	0.028	*n.s*.	2.31	0.023	*
AV vs audio-only	2.24	0.028	*n.s*.	2.44	0.017	*	2.67	0.009	*	2.09	0.040	*n.s*.	2.10	0.039	*
AV vs vib-only	1.59	0.117	*n.s*.	2.30	0.024	*	2.81	0.006	*	2.80	0.006	*	3.08	0.003	*

Statistical significances were evaluated using Holm’s method. FMS: Fast Motion Sickness scale; AV: audio-vibration; vib: vibration; *t*: *t*-value; non-adj. *p*: non-adjusted *p*-value; *n.s.*: not significant; *significant (*p* < 0.05).

The average total SSQ scores were 27.3 (*SD* = 34.0), 44.5 (*SD* = 34.0), 46.2 (*SD* = 38.0) and 51.2 (*SD* = 38.3) in the AV, no-AV, audio-only and vibration-only groups, respectively. A one-way ANOVA found no significant effect of the experimental group (*F* [3, 76] = 1.83, *p* = 0.18, *ω*^2^ = 0.02). The correlation coefficient between the 5^th^ FMS and total SSQ scores was 0.74 with a 95% confidence interval of 0.62–0.83.

**Figure 2:**
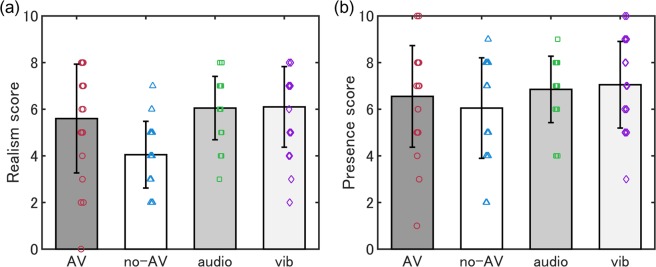
Realism (**a**) and presence (**b**) scores. The realism score is significantly lower in the no-AV group than in the other groups. AV: Audio-vibration; Vib: Vibration.

Figure 2 shows average realism and presence scores in each group. Realism scores were 5.6 (*SD* = 2.4), 4.1 (*SD* = 1.5), 6.1 (*SD* = 1.4) and 6.1 (*SD* = 1.8) in the AV, no-AV, audio-only and vibration-only groups, respectively. Presence scores were 6.6 (*SD* = 2.2), 6.1 (*SD* = 2.2), 6.9 (*SD* = 1.5) and 7.1 (*SD* = 1.9) in the same order, respectively. A one-way ANOVA found a significant effect of experimental group in the realism score (*F* [3, 76] = 5.68, *p* = 0.001; *ω*^2^ = 0.15) but not in the presence score (*F* [3, 76] = 0.97, *p* = 0.41; *ω*^2^ = 0.00). Multiple comparisons revealed that the realism score in the no-AV group was significantly lower than in the other three groups (*ts* [76] = 2.72, 3.51, and 3.59, *p* < 0.05).

**Figure 3:**
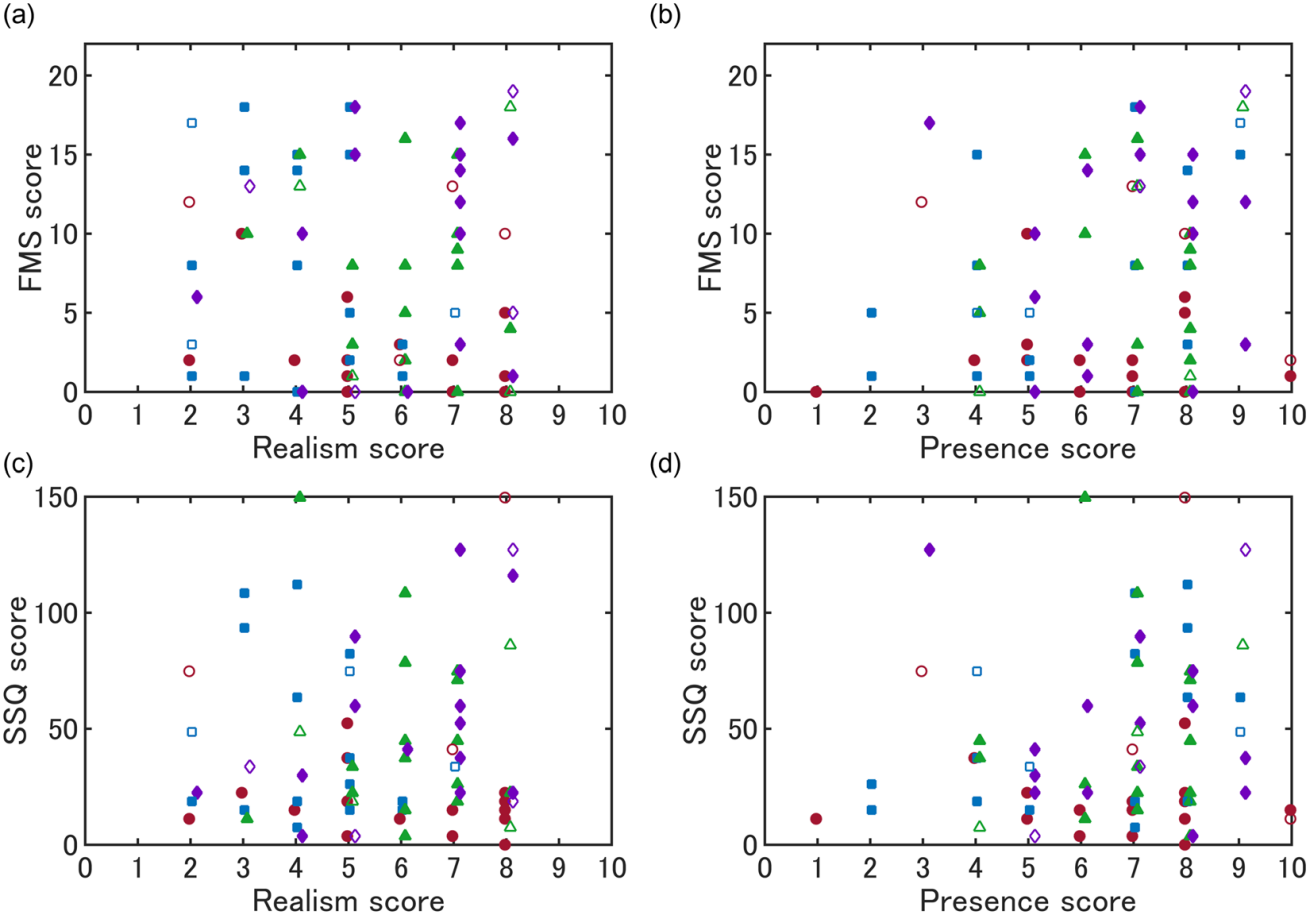
Scatter plots of presence and realism scores against VIMS scores (5^th^ FMS and total SSQ scores). Different markers indicate data of different experimental groups. Circle: AV group; Triangle: no-AV group; Square: audio-only group; Diamond: vibration-only group. Open markers indicate female data. There are no significant linear correlations between the variables. FMS: Fast Motion Sickness scale; SSQ: Simulator Sickness Questionnaire; AV: Audio-vibration.

Correlation coefficients were calculated to confirm the relationships between VIMS scores, realism and presence scores. Figure 3 shows the scatter plots of each pair. Correlation coefficients and 95% confidence intervals of the realism score were −0.03 (−0.25 and 0.19) for the 5^th^ FMS scores and 0.09 (−0.13 and 0.30) for the total SSQ scores, and those of presence score were 0.19 (−0.03 and 0.39) for the FMS scores and 0.16 (−0.06 and 0.37) for the SSQ scores.

To further identify the contributing factors of VIMS, we conducted multiple regression analyses for the 5^th^ FMS and total SSQ scores. To predict the VIMS score, we used the MSSQ score, SSQ score before the experiment (pre-SSQ), realism score, presence score, sex, car experience, motorcycle experience, VR experience, audio presentation and vibration presentation. The latter six variables were treated as binary dummy variables. In addition, we assumed that an interaction effect between presentation and vibration presentation would contribute to the VIMS scores. The results of the multiple regression analyses are given in Table 3. In the analysis for FMS, the coefficient of determination (*R*^2^) was 0.33, and the factors of MSSQ and presence were significant with beta coefficients = 0.36 and 0.24, respectively. The beta coefficient for the interaction between audio and vibration was −0.33 but not statistically significant. In the analysis for SSQ, *R*^2^ was 0.38, and the pre-SSQ and MSSQ were significant with beta coefficients = 0.33 and 0.39, respectively. The amplitude of the beta coefficient for the interaction between audio and vibration (−0.12) was lower than that of the FMS analysis (−0.32).

**Table 3:** The results of multiple regression analyses for the FMS and SSQ scores.

	FMS: *R*^2^ = 0.33					SSQ: *R*^2^ = 0.38				
	Regression coefficient	*t*	Beta coefficient	*p*		Regression coefficient	*t*	Beta coefficient	*p*	
(Intercept)	7.03	2.71		0.009		53.38	3.54		0.001	
Pre-SSQ	0.05	1.01	0.11	0.317		0.97	3.25	0.33	0.002	***
MSSQ	0.08	3.34	0.36	0.001	**	0.50	3.76	0.39	0.000	***
Sex	−0.50	−0.33	−0.03	0.746		−0.69	−0.08	−0.01	0.939	
Car	0.72	0.43	0.04	0.669		−8.43	−0.87	−0.09	0.387	
Bike	1.55	1.08	0.11	0.285		−12.37	−1.49	−0.15	0.142	
VR	−0.40	−0.28	−0.03	0.784		−8.50	−1.00	−0.12	0.321	
Realism	−0.45	−1.19	−0.15	0.237		1.67	0.76	0.09	0.450	
Presence	0.73	2.04	0.24	0.045	*	1.07	0.52	0.06	0.606	
Audio	0.29	0.15	0.02	0.879		−1.11	−0.10	−0.02	0.919	
Vibration	0.52	0.25	0.04	0.806		−3.24	−0.27	−0.04	0.792	
Audio × Vibration	−4.59	−1.59	−0.33	0.116		−14.45	−0.86	−0.17	0.392	

***p < 0.001, *p < 0.05. FMS*:* Fast Motion Sickness scale; SSQ: Simulator Sickness Questionnaire; MSSQ: Motion Sickness Susceptibility Questionnaire.

## Discussion

The present study investigated whether synchronised presentation of engine sound and vibration decreases VIMS while viewing a simulated motorcycle ride in a virtual environment. We showed that FMS scores were significantly lower in the AV group than in the other experimental groups that experienced only sound, vibration, or neither, which supports our hypothesis. In contrast to previous studies that investigated simple effects of sound or vibration, which did not reduce VIMS ^20,31,32^, this study demonstrated that simultaneous presentation of sound and vibration, where both were modulated by driving speed, can substantially reduce VIMS. Furthermore, a multiple regression analysis revealed that the MSSQ score was able to predict the severity of VIMS in our study.

Significant differences in the average FMS scores were observed even in the 2nd phase (2 minutes after the start of the visual stimulus), indicating that the effect of simultaneous sound and vibration presentation appears immediately after the start of the experiment. However, it should be noted that the FMS score increased as the time elapsed in the AV group. Although there was a significantly higher correlation between FMS in the 5th phase and SSQ scores (*r* = 0.74), SSQ scores did not significantly differ among the experimental groups. This discrepancy between the VIMS scales could be interpreted by considering that the SSQ evaluates a lot more aspects of motion sickness than FMS and by assuming that VIMS symptoms would decrease after one has finished a VR experience. Collectively, although generalizability of the technique to other VR scenes should be tested in future studies, the present results suggest that the presentation of engine sound and synchronised vibration suppresses the development of VIMS induced by a motorcycle driving scene.

This study, for the first time, showed that the presentation of engine sound and vibration substantially reduced VIMS while watching a driving scene. Whereas previous studies did not find a reliable effect of a simple presentation of sound or vibration on VIMS ^5,20,31,32^, using engine sounds and vibration that are necessarily entailed by motorcycle transportation, we showed that their combination had a significant reduction effect on VIMS. Adding environmental sounds, which have been used to investigate the role of sound in VIMS ^20,32^, would not be critical to decrease VIMS according to the sensory conflict theory since the environmental sounds mainly consist of unpredictable sounds, and many of them may not contain information directly linked to the visual flow. In other words, the noise of the town would not bring useful information to predict the motion of the first-person character. By contrast, engine sounds and vibration that sensitively reflect moving speed would help to predict incoming visual feedbacks, and the sensory integration would be more likely consistent with a “stored sensory integration”. Consistent with this idea, constant vibration, which is presented regardless of the visual scene, might not effectively reduce VIMS^31^. In addition to the reduction effect of audio-vibration presentation on VIMS, our experiments have demonstrated that adding either sound or vibration to a visual stimulus was insufficient to reduce VIMS, even though each of them is coupled with visual changes in a real world. The insignificant effect of the simple presentation of engine sounds is consistent with a previous result of a study with a car-driving simulator^5^. These findings may raise two possibilities; first, the effect of a simple presentation of sound or vibration is not strong enough to reduce VIMS, and the effect is accumulated and exceeds a certain threshold when they are presented at the same time. This hypothesis is not limited to sound and vibration but includes other types of sensory information, for example, tactile stimulation by airflow. Second, a simple presentation of sound or vibration does not work for reducing VIMS at all because they are tightly coupled with each other in reality and missing one could create an inconsistency in sensory integration. These possibilities are not mutually exclusive and should be further examined in future studies.

The current study also investigated the relations between perceived realism and presence and VIMS. The correlation analysis did not find any reliable correlation coefficients among the factors (*r* = −0.03–0.19). The findings with regard to the relation between the feelings of presence in the visual scene and VIMS are inconsistent with those in previous studies. A recent review^38^ has shown that there may be a negative relationship between presence and VIMS; when the perceived presence in a VR increases, VIMS decreases. Moreover, negative correlations between presence and VIMS scores have been found in Nichols, *et al*.^39^ and Milleville-Pennel and Charron^23^. However, D’Amour, *et al*.^31^, who investigated VIMS during watching a bicycle-ride scene, did not found a significant correlation between the presence and FMS scores and a positive correlation (*r* = 0.26) between the realism and FMS scores. Furthermore, in contrast with the review’s implication, our multiple regression analysis showed a positive contribution of presence score to the FMS score (beta = 0.24). The reasons for these divergent results may be caused by inconsistency in VR scenes or tasks, types of VR device^38^, the severity of VIMS and statistical designs (e.g., whether the correlation analysis included inter-group data or not, and presence/realism was manipulated or co-variated; statistical power related to sample size largely differs between studies). It might also be a problem to explore the relations between the subjective feelings to a VR scene and VIMS solely based on simple correlation analyses because influences by other factors cannot be excluded. The current findings at least suggest that the perceived presence may interact with the mechanism that induces VIMS, although it is not always linearly correlated with VIMS scores.

It is worth to mention two other findings. First, the presentation of sound and/or vibration increased the realism score (*ω*^2^ = 0.15) but not the presence score (*ω*^2^ = 0.00). These results suggest the presence of the VR scene, “the feeling of being there in the virtual scene”, may not be increased by just presenting engine sounds and vibrations of a motor cycle, whereas the realism, “how real the virtual scene is”, is more sensitive to addition or interaction of sensory information. Consistent with our results, D’Amour *et al*.^31^ did not find any significant differences in presence scores among experimental conditions, while one of their experimental condition using air flow and vibration successfully reduced VIMS. It is, nevertheless, possible that a ceiling effect might have occurred in the presence scores, and thus the scores did not differ among the experimental groups because our study used an HMD, which more likely provides us an immersive experience than display-based devices. Second, the MSSQ scores had significant estimated contributions to both FMS and SSQ scores (beta = 0.35 and 0.36), whereas the SSQ score before the experiment had a significant estimated contribution to the SSQ score after the experiment (beta = 0.36). Other factors investigated (sex, car experience, motorcycle experience and VR experience) did not contribute to the severity of VIMS (beta = −0.15–0.11). Consistent with Dennison and D’Zmura^40^, where MSSQ and SSQ scores were positively related (*r* = 0.67), we found a significant correlation (FMS: *r* = 0.41; SSQ: *r* = 0.40). Note, however, other VIMS studies using the MSSQ have not found a correlation between the MSSQ and VIMS measures^41–43^. Therefore, further studies are needed to confirm that the MSSQ is related to VIMS. Our findings suggest that adding sound or vibration information likely increases the perceived realism of a VR driving scene, and self-reported vulnerability to motion sickness can predict the severity of VIMS symptoms in a motorcycle driving scene, even when other factors are taken into account.

The present study has several limitations. First, this study did not include a sufficient number of female participants; second, the length of the VR experience (5 min) was relatively short; and third, we did not examine the effect of synchronization among the sensory information on VIMS. Nevertheless, the present study provided strong evidence that the combined presentation of sound and vibration significantly reduces VIMS. Future studies should include a larger number of female participants. They could further consider experimental conditions, where sound and vibration are not synchronous with one another or driving speed. Longer presentation of the VR experience would also be useful to remove a possible floor effect of VIMS symptoms and thus make statistical analyses more rigorous. In addition, variables included in the multiple regression model in our study were selected rather in a bottom-up way, and therefore should be refined based on the current results in future studies.

In conclusion, the present study demonstrated a reduction effect of the synchronous presentation of sound and vibration on VIMS. This result may be explained by the sensory conflict theory. In addition, we found that adding sound and vibration increased realism but did not affect presence, suggesting that VIMS can be suppressed without deteriorating the VR experience when using a vibration device. If vibrations from a handlebar or an HMD, instead of the seat vibration, also reduces VIMS, the application of the proposed methods would become further easier. The results of this motorcycle simulation study may also pertain to other driving simulations, where speed-dependent engine sounds and vibrations also play a role. In accordance with sensory conflict theory, we would also expect similar effects for sounds and vibrations that are produced by roads, tracks, or air resistance as well as engine motion itself, that are usually proportional to engine speed. The application to human locomotion in VR scenes may not be the same but worthwhile trying.

## Materials and Methods

### Participants

Eighty healthy students (16 females, 64 males) participated in the study (*Mean age* = 21.3 years, *SD* = 1.2). This study used a between-participants design to avoid the adaptation effect ^25,26^ on the VR environment and VIMS. The participants were randomly assigned to the audio-vibration (AV), no-audio-vibration (no-AV), audio-only, or vibration-only groups (4 females in each group). All participants were recruited via a university mailing list, and they had no signs of sickness, sleep problems, or sensory-motor problems and provided written informed consent before the study. The study was approved by the ethics committee of Shizuoka University (No. 18–14) and was conducted following the approved guidelines and regulations.

### Stimuli and apparatus

**Figure 4:**
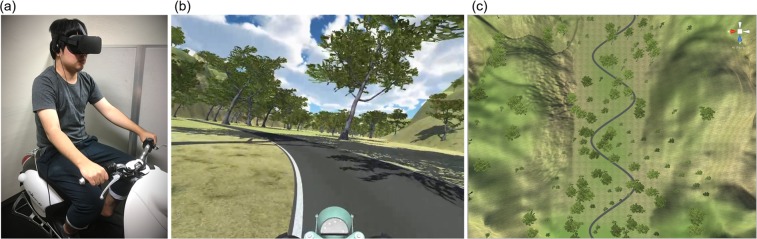
Experimental settings (**a**), presented visual scene (**b**) and winding road in the virtual reality environment (**c**). The individual in the 3a is one of the co-authors who provided informed consent to publish the image.

The experiment was conducted in an indoor room. Participants watched a visual stimulus through an HMD when sitting on a chassis of a scooter that was removed of an actuator and grabbing the handgrips (Fig. 4a). A vibration device (Vt7, Acouve, Inc., Japan) was equipped under the seat. The scooter’s tires did not touch on the floor, and the body could not lean to the lateral sides. The HMD (Oculus Rift, Facebook Technologies, LLC., USA), which offers the visual resolution of 2160 × 1200 in the 110° field of view, was used to display a driving scene. The driving scene was controlled by Unity. The refresh rate of the display was 90 frames per second. The visual stimulus simulated a driving scene on a winding road from a first-person viewpoint (Fig. 4b). The winding road, which was demonstrated to induce VIMS more than a straight road^10^, was selected to elicit VIMS. The bird view of the road is shown in Fig. 4c. A driving scene, which took 54 seconds, was repeated seamlessly in a 5-minute experiment. The time of experiencing the driving scene was determined based on the results of a preliminary experiment. A head-tracking function was available during the experiment.

Figure 4 In reality, driving a curved road causes the inclination of the motorcycle body. To simulate this motion in the virtual environment, we used equation (1) below:

1 $$\theta ={\tan }^{-1}\left(\frac{{v}^{2}}{ag}\right)$$

where *g* = the gravity acceleration, *v* = the speed of the motorcycle, *a* = the radius of motorcycle displacement and *θ* = the inclination of the motorcycle body relative to the road.

**Figure 5:**
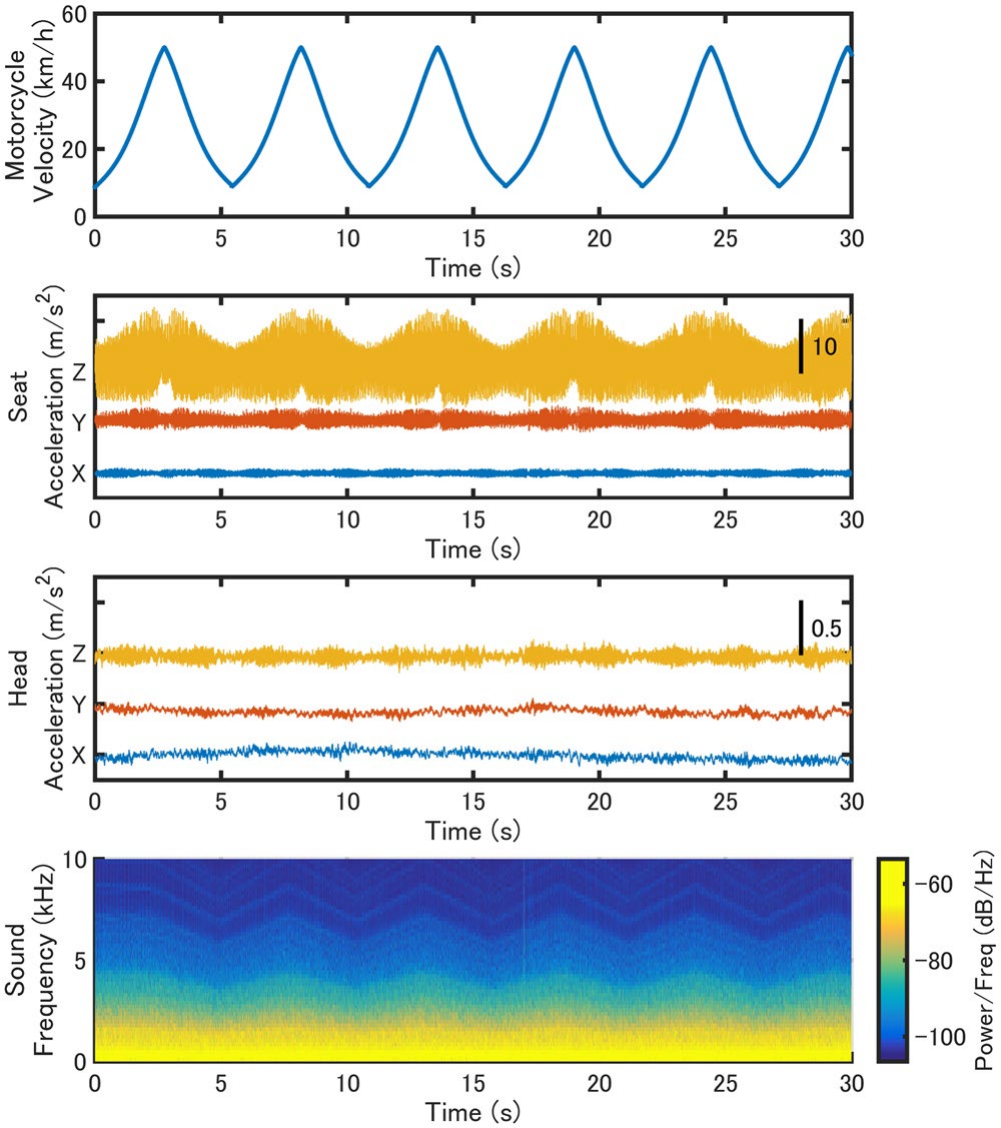
The speed of motorcycle in the virtual reality environment, frequency spectrogram of the presented engine sound and accelerations measured on the seat and head-mounted display.

Engine sounds were created using a motorcycle engine sound synthesis software (Sound Design Lab, LLC., Japan) and were presented by earphones. The speed of the motorcycle changed from 10 to 50 km/h. The pitch of the engine sound was modulated with the driving speed, but the amplitude of the sound did not change. The vibration device (Vt7) attached under the seat could present vibrations with frequencies from 16 to 150 Hz. The frequency of the vibration was modulated based on the frequency of the sound. Figure 5 shows the speed of the motorcycle (top panel), the accelerations measured on the seat (second panel) and forehead (third panel) and the frequency spectrogram of the presented engine sound (bottom panel) in the VR environment. The mean sound pressure level (*L*_*p*_) of the engine sound in 30 seconds at the earphone was 91.3 dB in the AV and audio-only groups, its loudness level (*L*_*A*_) was 96.8 dB, which was measured by a sound level meter (6226, ACO, Co., Ltd, Japan), the root mean square (RMS) of the total accelerations (RMS of the vector sum of the three components) in 30 seconds was 4.56 (*Max* = 13.02) m/s^2^ on the seat and 0.15 (*Max* = 0.25) m/s^2^ on the forehead. The acceleration of the seat was measured without a participant sitting on it, and the acceleration on the forehead was measured with a representative participant sitting on the seat. The participants wore earphones, except in the vibration-only group, where they wore a noise cancelling headphone (QuietComfort 35, Bose, Inc., USA) to reduce possible sounds induced by vibration. The RMS of the acceleration values was similar to that in the study by D’Amour, *et al*.^31^ (Seat = 4.25 m/s^2^, Head = 0.10 m/s^2^).

First, we evaluated the proneness of motion sickness using the MSSQ^44^ based on the experience of motion sickness. The possible maximum MSSQ score is 427.68. The mean MSSQ scores were 22.9 (*SD* = 20.1), 21.5 (*SD* = 27.0) and 77.2 (*SD* = 38.6) in the studies by Dennison and D’Zmura^40^, Bos, *et al*.^42^ and Bos^41^, respectively. In the present study, the average MSSQ scores were 77.2 (*SD* = 30.2), 80.5 (*SD* = 25.7), 70.5 (*SD* = 18.9) and 91.3 (*SD* = 32.9) in the AV, no-AV, audio-only and vibration-only groups, respectively.

Before watching the visual stimulus through the HMD, the participants answered the SSQ to confirm that they did not report symptoms prior to exposure. In the main observation phase, participants watched the presented stimulus for 5 minutes and were instructed not to move the handlebar and not to lean their trunk. When the participants wore the HMD, the virtual motorcycle waited to start with idling engine. The motorcycle was kept driving from the start to the end of the observation phase, and engine sounds and/or vibrations were provided to the participants according to the experimental group type. Sounds and vibration were not provided to the no-AV group. The participants answered the FMS by saying the number (0–20) when they found the word “Answer” in the centre of the screen. The FMS was evaluated every 1 minute, and therefore five times in total. The display of “Answer” disappeared after 3 seconds from the presentation, and all participants appropriately answered FMS without missing them. Immediately after the 5^th^ evaluation of FMS, the visual stimulus was stopped, and the participants took off the HMD and answered SSQ. They then took a short rest and answered subjective reality and presence (11-point scale from 0 to 10) of the visual stimulus presented in the HMD, in addition to the various experience, to comprehensively identify the factors of VIMS. The questionnaire items (realism: *the feeling of being there in the virtual scene that you experienced*; presence: *the feeling of how real the virtual scene that you experienced was*) were created based on previous studies^23,31^.

We evaluated motion sickness using two different questionnaires. SSQ, a standardised questionnaire that covers extensive symptoms of VIMS^35^, consists of 16 items with 4-point scales (0 [absent] to 3 [severe]), and the possible maximum total score (SSQ-TS) is 235.62^45^. Although SSQ has three subscales (N: nausea; O: oculomotor; D: disorientation), we analysed only the total score to simplify the analysis. The mean SSQ scores assessed before the experiment were 11.4 (*SD* = 11.7), 9.7 (*SD* = 14.1), 13.5 (*SD* = 13.3) and 6.0 (*SD* = 10.0) in the AV, no-AV, audio-only and vibration-only groups, respectively. We did not calculate the difference between the scores at the baseline and after the observation phase. VIMS during watching the driving scene was evaluated by FMS. FMS focuses on nausea, general discomfort and stomach problems but asks participants to ignore other symptoms such as nervousness, cold sweat, boredom, fatigue and drowsiness^9^, which was instructed to the participants prior to the observation phase. Participants answered the degree of VIMS by verbally answering the number (0 [*no sickness at all]* to 20 [*frank sickness*]). The correlation coefficients between the SSQ total score and FMS score were *r* = 0.79 in the study by Keshavarz and Hecht^9^, *r* = 0.71 in the study by Keshavarz and Hecht^30^ and *r* > 0.61 in the study by D’Amour, *et al*.^31^.

The participants also rated the realism and presence of the visual stimulus (0–10). Before the rating, the participants were explained that realism means the feeling that the virtual environment is a part of real life, and presence means the feeling of being there in the virtual scene^31,46^. Furthermore, they answered the frequency of their daily use of a car or motorcycle, and they stated whether they had ever experienced a VR device such as Oculus Rift.

The participants could quit the experiment whenever they wanted and by whatever reasons to avoid a severe motion sickness that affects daily activities after the experiment. Six participants quit watching visual stimulus; the number of dropouts was two, one and three in the no-AV, audio-only and vibration-only groups, respectively. Their data were not excluded from the analysis, and the FMS that could not be answered were scored as the FMS rating immediately before the quitting, as in the study by D’Amour, *et al*.^31^.

## Data Availability

The datasets generated and/or analysed during the current study are available from the corresponding author on reasonable request.
